# Structure–Activity Relationship for Inflammasome Inhibition by Thiomuscimol

**DOI:** 10.3390/ijms27167235

**Published:** 2026-08-13

**Authors:** Marisa J. Anderson, Wendy P. Loomis, Andreas B. den Hartigh, Bente Frølund, Susan L. Fink

**Affiliations:** 1 Department of Laboratory Medicine and Pathology, University of Washington, Seattle, WA 98195, USA; 2 Department of Drug Design and Pharmacology, Faculty of Health and Medical Sciences, University of Copenhagen, 2100-Ø Copenhagen, Denmark

**Keywords:** inflammasome, pyroptosis, thiomuscimol, structure–activity relationship, gasdermin D, ASC, caspase-1, innate immunity

## Abstract

Inflammasomes are central mediators of innate immune defense but can also drive pathological inflammation and pyroptotic cell death in numerous diseases. While several small-molecule inhibitors have been described, many selectively target individual inflammasomes or act through the adaptor protein ASC, leaving ASC-independent pathways unaffected. We previously identified thiomuscimol as a broad-spectrum inflammasome inhibitor that blocks both ASC-dependent and ASC-independent activation, although the structural basis for this activity remains unclear. Here, we examined the structure–activity relationship of thiomuscimol using related compounds and synthetic analogs. In primary macrophages, inflammasome activation and pyroptosis were assessed by live cell imaging of ASC speck formation, gasdermin D-mediated dye uptake, and cellular ATP levels. Structurally related sulfur-containing molecules, including taurine and isothiazole, failed to inhibit inflammasome activation, indicating that neither the sulfur in an electron-rich environment nor the heterocyclic scaffold confer activity. Replacement of the primary amine with a carbonyl group abolished activity, whereas substitution with a secondary amine preserved inhibitory potency comparable to thiomuscimol. Incorporation of the amine into an annulated piperidine ring reduced potency and revealed sensitivity to the precise positioning of the amine within the ring. Together, these findings identify key structural features required for thiomuscimol-mediated inflammasome inhibition and provide a framework for future studies to define its mechanism of action and guide the development of improved inhibitors.

## 1. Introduction

Inflammasomes are multiprotein signaling complexes that are essential in the innate immune system, acting as the first line of defense against pathogens or environmental factors posing as immune threats [1]. Inflammasome sensor proteins detect molecular patterns and cellular perturbations leading to recruitment and activation of pro-caspase-1, either directly via caspase recruitment domain (CARD) interactions, or through pyrin domain (PYD) interactions with the adapter protein ASC [2]. The activated caspase-1 protease cleaves the pore-forming protein gasdermin D, separating the inhibitory domain from the pore-forming domain, which translocates to the plasma membrane and oligomerizes to form pores, leading to pyroptotic cell death [3]. Activated caspase-1 also cleaves the precursors of interleukin-1β (IL-1β) and IL-18 to produce active inflammatory cytokines, which are released via gasdermin D pores.

Although inflammasomes are critical for innate immune defense, they also play a role in the pathogenesis of multiple different disease states through initiating inflammation and cell death [4]. For example, dysregulated inflammasome responses to infection during sepsis contribute to life-threatening organ dysfunction [5,6]. Inflammasomes and pyroptosis are implicated in other disease states, affecting a variety of organ systems, such as neurological, respiratory, cardiovascular, and digestive diseases [7,8]. The central role of inflammasomes in many disease processes makes them attractive targets for therapeutic intervention. As a result, the discovery and characterization of novel inflammasome inhibitors has been an area of active investigation [9,10].

The pathogenesis of complex diseases often involves the activation of multiple inflammasomes [11]. Given the overlapping and compensatory nature of inflammatory signaling networks, interventions designed to block multiple inflammasomes simultaneously could be of great therapeutic value. One strategy for inhibiting multiple inflammasomes is to target the shared adaptor protein ASC, a mechanism employed by the small-molecule inhibitor MM01 [12]. A limitation of this approach is that some inflammasome sensors can activate caspase-1 independently of ASC [13,14], thereby bypassing ASC-targeted inhibition.

We previously identified the small molecule thiomuscimol as a novel broad-spectrum inflammasome inhibitor with activity against the NLRP1b, NLRP3, NLRC4, pyrin, and AIM2 inflammasomes [15]. Thiomuscimol inhibited both ASC-dependent and ASC-independent inflammasome activation, consistent with a mechanism distinct from that of other characterized inflammasome inhibitors. However, the molecular mechanism responsible for this unique activity remains incompletely understood. Here, we examine a series of thiomuscimol analogs to characterize the structure–activity relationship and define the chemical features necessary for inflammasome inhibition.

## 2. Results

**Figure 1:**
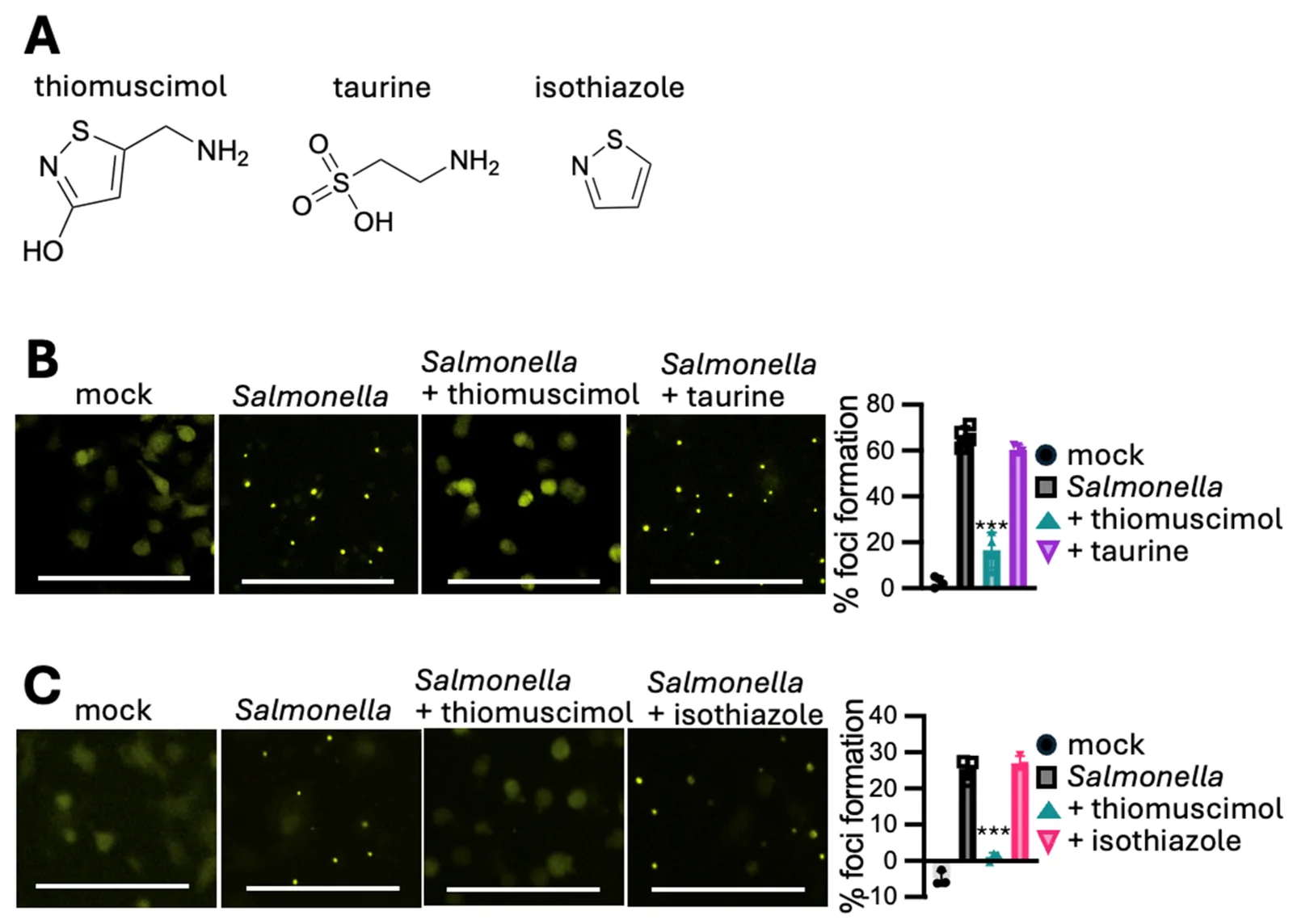
Sulfur analogs of thiomuscimol like taurine and isothiazole do not inhibit inflammasome activation. Structures of thiomuscimol, taurine, and isothiazole (**A**). Bone marrow-derived macrophages (BMDMs) expressing ASC–citrine were infected with *Salmonella* at an MOI of 10 to stimulate pyroptosis through the NLRC4 inflammasome in the presence of the indicated compounds at 1 mM and ASC foci formation was assessed (**B**,**C**). Scale bars represent 100 μm. Data are means +/− SD, *n* = 3 or 4 replicate wells, and are representative of at least two independent experiments. Stars (*) indicate statistical significance against *Salmonella*. *** *p* < 0.001 (one way ANOVA, with Dunnett’s multiple comparisons test).

We previously found that muscimol prevents pyroptosis-associated plasma membrane rupture, without affecting any upstream portions of the pyroptotic cascade [16]. Muscimol differs from thiomuscimol only by a single heteroatom substitution within the five-membered ring, containing an oxygen in place of the sulfur present in thiomuscimol. Our observation that muscimol has no activity to prevent inflammasome formation, in contrast to thiomuscimol [15], suggests that the presence of sulfur in the heterocyclic ring may be an essential molecular feature for inflammasome inhibitory activity. We thus sought to determine whether other similar sulfur-containing compounds prevent inflammasome activation. Taurine is a linear amino sulfonic acid, which, like thiomuscimol, contains a primary amine and sulfur atom. Although taurine exists in a distinct electronic and steric environment (Figure 1A) and is more readily deprotonated because of its greater acidity, it shares GABA-A receptor agonist activity with thiomuscimol [17].

To determine whether taurine also shares the ability to block inflammasome activation, we utilized live cell microscopy of primary macrophages expressing the inflammasome adapter ASC fused with the fluorescent protein citrine [18]. In uninfected cells, the ASC–citrine fusion protein is diffusely distributed throughout the cytoplasm but localizes into a single large focus or “speck” upon NLRC4 inflammasome activation with *Salmonella* infection. Consistent with our previous results [15], thiomuscimol prevented ASC foci formation (Figure 1B). In contrast, ASC foci formation was unaffected by an equimolar concentration of taurine (Figure 1B). We confirmed that thiomuscimol also blocks downstream effects in the pyroptotic cascade, inhibiting both IL-1β release (Supplemental Figure S1A) and the release of lactate dehydrogenase (LDH), a large cytoplasmic protein liberated by pyroptotic plasma membrane rupture (Supplemental Figure S1B). In contrast to thiomuscimol and consistent with its lack of effect on ASC foci, taurine did not prevent IL-1β release (Supplemental Figure S2A), nor did it inhibit LDH release (Supplemental Figure S2B). These findings indicate that not all sulfur-containing compounds inhibit inflammasome activation or downstream pyroptotic effects and suggest that the heterocyclic ring structure may also contribute to activity.

We next tested isothiazole, which shares the identical five-membered aromatic ring structure with thiomuscimol, containing adjacent nitrogen and sulfur atoms, but without the acidic OH group (Figure 1A). Unlike thiomuscimol, isothiazole did not prevent ASC foci formation during *Salmonella* infection (Figure 1C), nor did it inhibit IL-1β release (Supplemental Figure S3A) or LDH release (Supplemental Figure S3B). These results suggest that the minimal sulfur-containing heterocyclic scaffold is not sufficient for activity and the additional substituents may also play a role. Taken together, these data indicate that neither the presence of a sulfur as part of a sulfonic acid nor the five membered ring alone are sufficient to recapitulate thiomuscimol’s ability to inhibit inflammasome activation.

**Figure 2:**
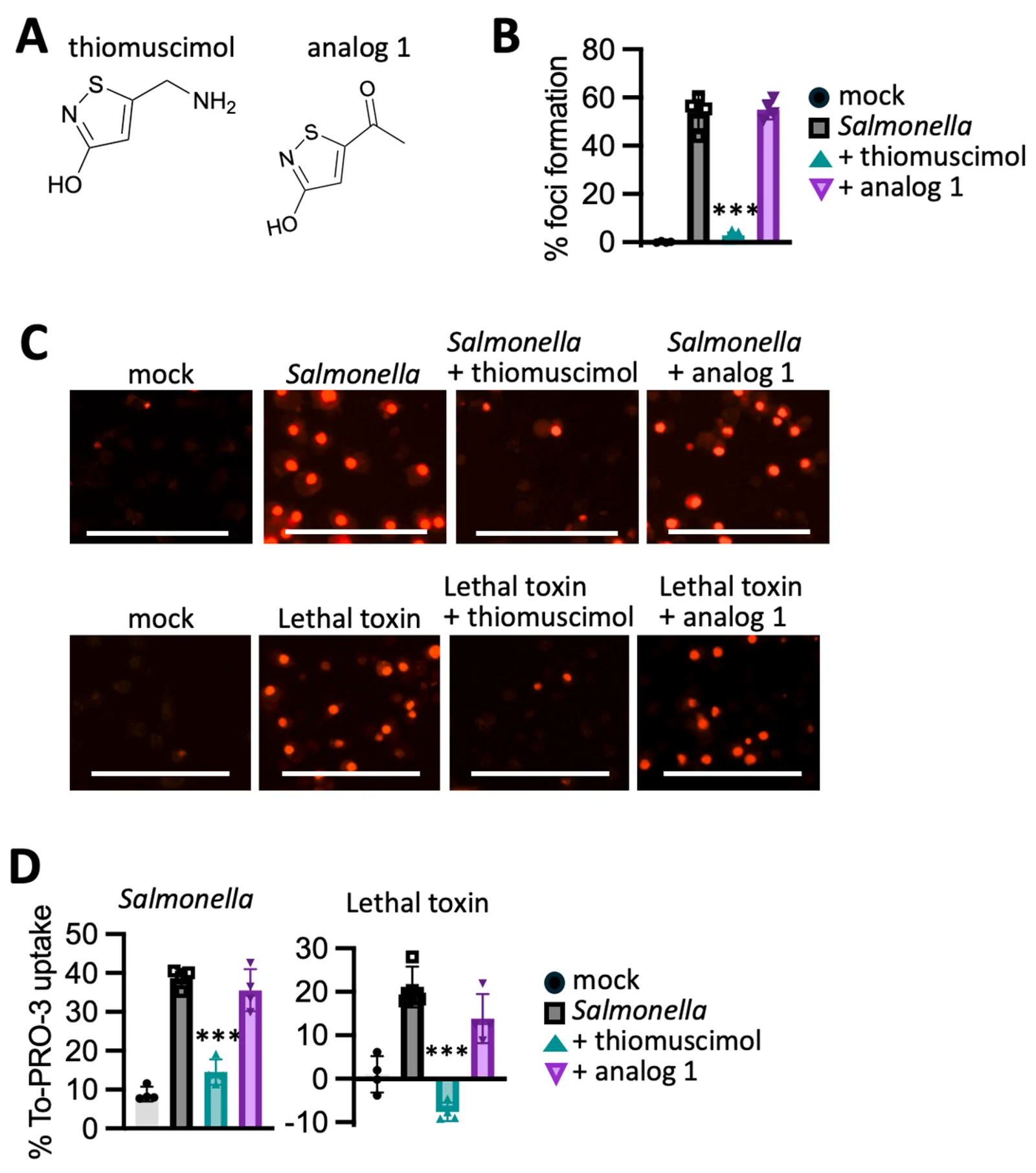
Substitution of the primary amine with a carbonyl group abolishes inflammasome inhibition. Structures of thiomuscimol and analog 1 (**A**). ASC–citrine-expressing BMDMs were infected with *Salmonella* at an MOI of 10 to stimulate pyroptosis through the NLRC4 inflammasome in the presence of the indicated compounds at 1 mM and ASC foci formation was assessed (**B**). BMDMs were infected with *Salmonella* or treated with lethal toxin to induce pyroptosis in the presence of the indicated compounds at 1 mM and To-PRO-3 uptake was measured (**C**,**D**). Scale bars represent 100 μm (**C**). Data are means +/− SD, *n* = 3 or 4 replicate wells, and are representative of at least two independent experiments. Stars (*) indicate statistical significance against *Salmonella* or lethal toxin, where indicated. *** *p* < 0.001 (one way ANOVA, Dunnett’s multiple comparisons (**B**,**D**)).

One of the ring substituents that could contribute to thiomuscimol’s activity is the primary amine (Figure 2A). Thus, we examined the effect of replacing the primary amine with a carbonyl group (analog 1, Figure 2A) and found that this analog failed to inhibit ASC foci formation (Figure 2B), indicating loss of an effect on inflammasome activation. NLRP1b and NLRC4 both activate caspase-1 to cleave gasdermin-D as a part of the pyroptotic cascade progression. Gasdermin D pores mediate entry of small molecules, including the membrane-impermeable fluorescent nucleic acid stain, To-PRO-3 [19]. Consistent with our previous findings [15], thiomuscimol blocked gasdermin D-mediated To-PRO-3 uptake in response to both NLRC4 inflammasome activation with *Salmonella* infection and NLRP1b inflammasome activation with anthrax lethal toxin (Figure 2C). In contrast, analog 1 had no effect on To-PRO-3 uptake in response to either stimulus (Figure 2C), indicating loss of activity against inflammasome activation.

**Figure 3:**
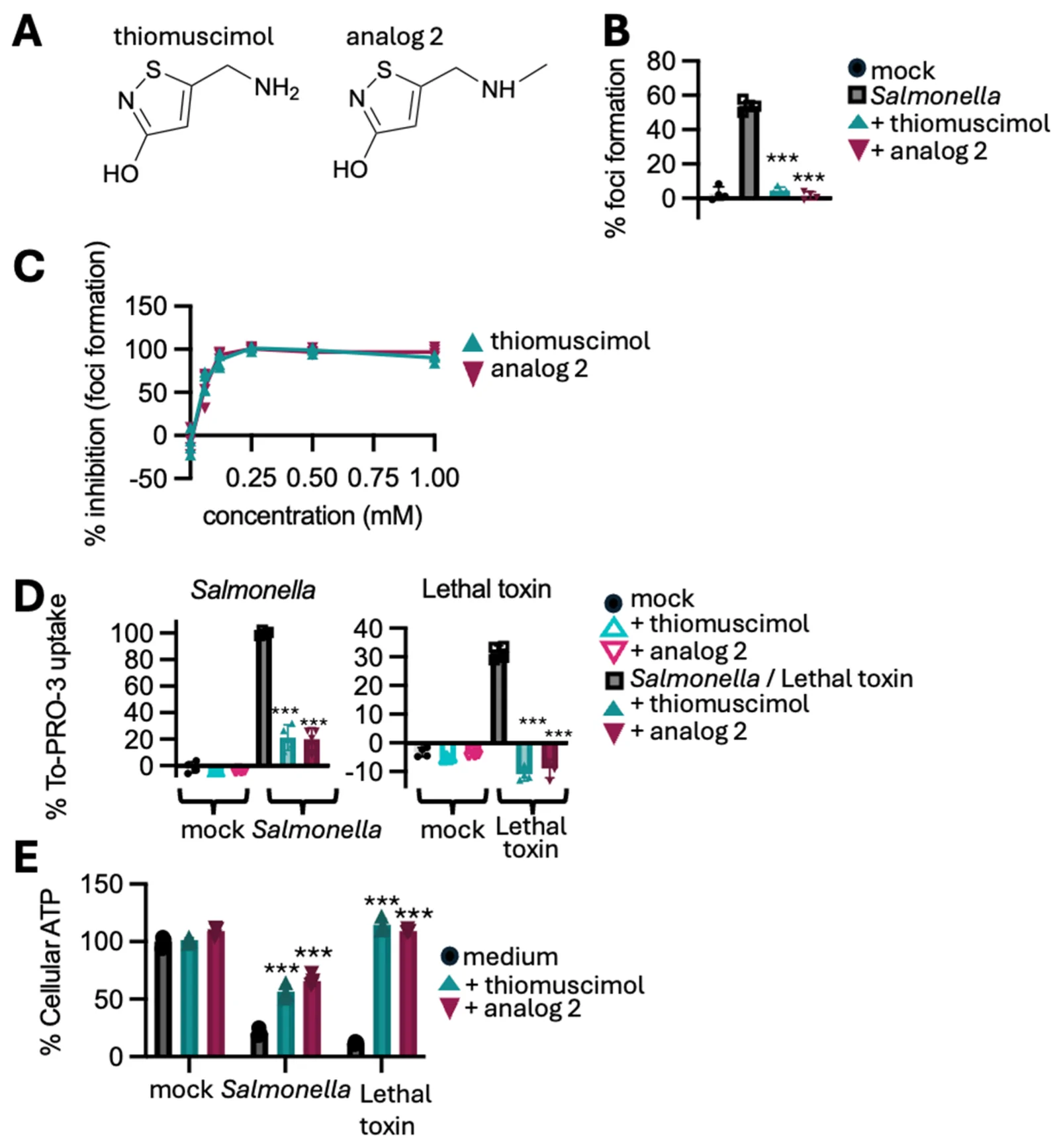
Substitution of a primary to secondary amine preserves inflammasome inhibition. Structures of thiomuscimol and analog 2 (**A**). BMDMs were infected with *Salmonella* at an MOI of 10 or treated with lethal toxin to induce pyroptosis in the presence of the indicated compounds. Inhibitor concentrations are 0.0625, 0.125, 0.25, 0.5, and 1 mM (**C**), or 1 mM (**B**,**D**,**E**). ASC foci formation was assessed (**B**) and inhibition at the indicated concentration was calculated relative to vehicle control (**C**). To-PRO-3 uptake during *Salmonella* infection or lethal toxin treatment was measured in the presence of the indicated compounds (**D**). Cellular ATP content was measured in the presence of the indicated compounds and pyroptosis inducer (**E**). Data are means +/− SD, *n* = 3 or 4 replicate wells, and are representative of at least two independent experiments. Stars (*) indicate statistical significance against *Salmonella* or lethal toxin, where indicated. *** *p* < 0.001 (one way ANOVA, with Dunnett’s multiple comparisons test (**B**,**D**) or Tukey’s multiple comparisons test (**E**)).

We next examined replacement of the primary amine to a secondary amine (analog 2, Figure 3A) and found that this substitution permitted ASC foci inhibition (Figure 3B). We tested analog 2 over a range of concentrations and found that it blocked ASC foci formation, exhibiting similar activity under the tested concentrations as thiomuscimol (Figure 3C). Analog 2 had a similar IC50 to thiomuscimol, with an IC50 of 58.47 μM for analog 2, and 45.12 μM for thiomuscimol. Like thiomuscimol, analog 2 also inhibited IL-1β release (Supplemental Figure S4A). To determine whether analog 2 also blocked gasdermin D pore formation subsequent to inflammasome activation, we assessed To-PRO-3 uptake. Similar to thiomuscimol, analog 2 inhibited To-PRO-3 entry in response to both NLRC4 inflammasome activation with *Salmonella* infection and NLRP1b inflammasome activation with anthrax lethal toxin (Figure 3D). In addition to allowing small dye entry, gasdermin D pores mediate loss of cytosolic ATP, leading to cessation of metabolic activity [20]. We confirmed our previous finding that thiomuscimol at least partially restored cellular ATP content in response to both *Salmonella* infection and anthrax lethal toxin treatment (Figure 3E). Analog 2 demonstrated a similar ability to rescue ATP content in response to both stimuli (Figure 3E), suggesting that like thiomuscimol, analog 2 restores cellular viability. Neither thiomuscimol nor analog 2 caused a significant decrease in ATP content with the compounds alone, indicating that the observed effects were not due to cytotoxicity. Additionally, neither thiomuscimol (Supplemental Figure S1B) nor analog 2 induced LDH release when presented alone, and both inhibited LDH release during pyroptotic conditions (Supplemental Figure S4B). Together, these results indicate that the replacement of a primary amine with a secondary amine does not appreciably alter the molecule’s ability to inhibit upstream steps in the pyroptotic cascade.

**Figure 4:**
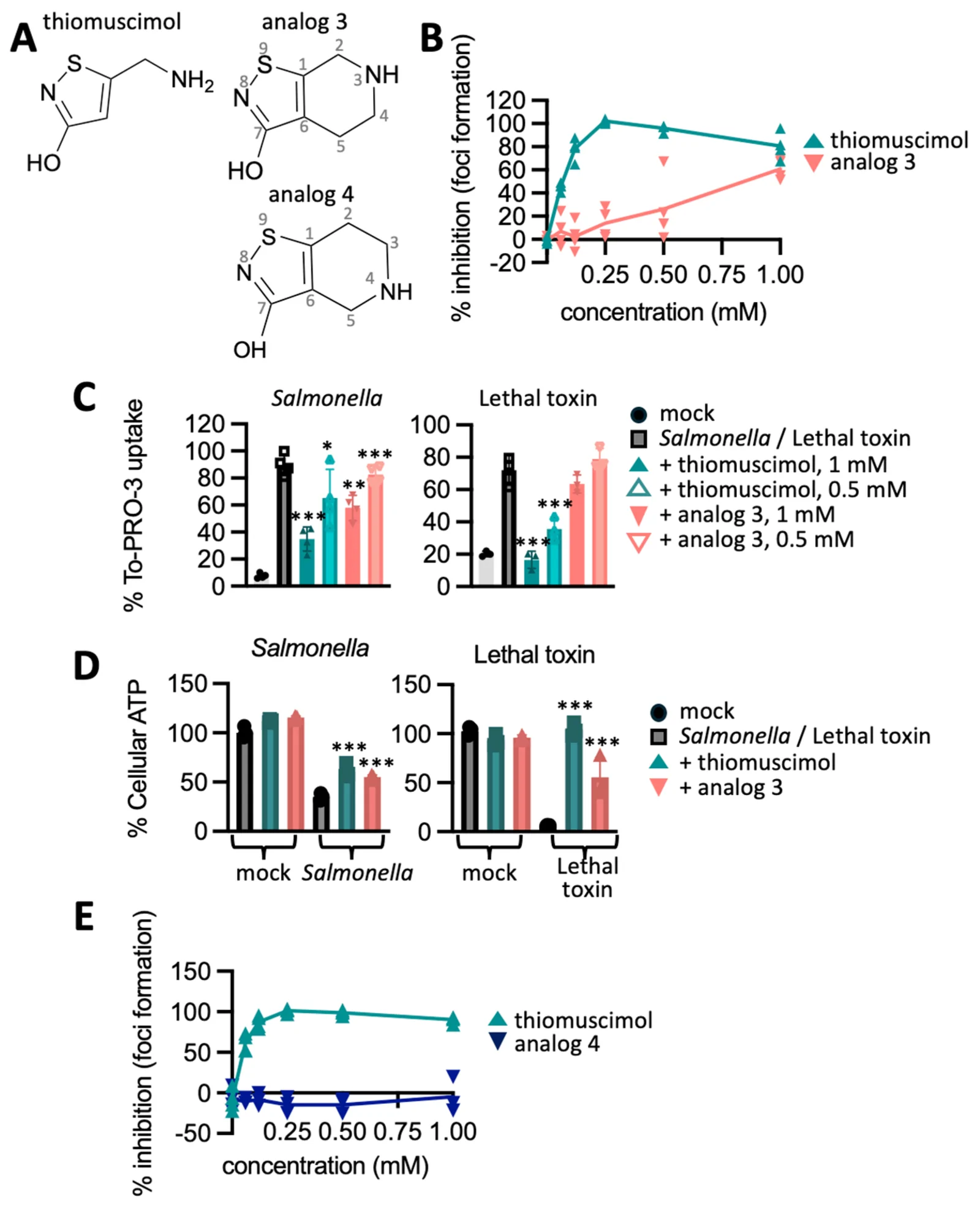
Cyclization alters thiomuscimol activity, and secondary amine position alters effects. Structures of analogs (**A**). BMDMs were infected with *Salmonella* at an MOI of 10 to induce pyroptosis in the presence of the indicated compounds. Inhibitor concentrations are 0.0625, 0.125, 0.25, 0.5, and 1 mM (**B**,**E**). ASC foci formation was assessed and inhibition at the indicated concentration was calculated relative to vehicle control (**B**). To-PRO-3 uptake during *Salmonella* infection or lethal toxin treatment was measured (**C**). Cellular ATP content was measured in the presence of the indicated compounds and pyroptosis inducer (**D**). Data are means +/− SD, *n* = 3 or 4 replicate wells, and are representative of at least two independent experiments. Stars (*) indicate statistical significance against *Salmonella* or lethal toxin, where indicated. * *p* < 0.05, ** *p* < 0.01, *** *p* < 0.001 (one way ANOVA, with Dunnett’s multiple comparisons test (**B**,**C**), or Tukey’s multiple comparisons test (**D**)).

Finally, we examined incorporating the amine into an annulated piperidine group, retaining the presence of a secondary amine as in analog 2 (analogs 3 and 4, Figure 4A). We found that analog 3 inhibited ASC foci formation similarly to thiomuscimol at the highest concentration tested, but had an observed decrease in activity at 0.5 mM and loss of activity at 0.25 mM and lower (Figure 4B). This difference was reflected in the increased IC50 for analog 3 (81.24 μM compared to 45.12 μM for thiomuscimol). Similarly, analog 3 inhibited To-PRO-3 uptake in response to both *Salmonella* infection and anthrax lethal toxin treatment, with slightly reduced efficacy in comparison to thiomuscimol (Figure 4C). Analog 3 also demonstrated restoration of ATP content in pyroptotic cells, with no observed cytotoxicity (Figure 4D). The reduced activity at tested concentrations for analog 3 could indicate that the flexibility in the carbon chain containing the amine group may be important for thiomuscimol’s activity. We observed that the position of the secondary amine within the piperidine ring was critical for activity; when the position of the amine was changed, from that in analog 3 to analog 4, there was no inhibition of ASC foci formation (Figure 4E). Additionally, when the amine was moved further away from the five-membered ring and included as part of a non-annulated piperidine ring-system (analog 5), there was no inhibition of ASC foci formation (Supplemental Figure S5). These results indicate that while an annulated piperidine substitution permits altered activity, the position of the secondary amine group within the structure is critical to retain the ability to inhibit inflammasome activation and pyroptosis.

## 3. Discussion

Excessive inflammasome activity that drives subsequent inflammation and pyroptotic cell death is an increasingly recognized contributor to disease progression and pathogenic consequences. Given that inflammasomes are implicated in a growing number of disease states, the development of small molecule antagonists is an area of great interest and therapeutic importance. Many of the inhibitors that have been recently identified, such as DVF890 or NT-0796 [21,22], target only the NLRP3 inflammasome. Although inhibition of specific inflammasomes may be beneficial, particularly in disease states where a single inflammasome is strongly implicated in pathogenesis, the significant crosstalk within immune pathways could necessitate an inhibitor that acts more broadly for therapeutic application. Thiomuscimol is uniquely able to block activation of multiple inflammasomes via an ASC-independent mechanism, and this study identified several molecular determinants governing this novel activity.

Our previous observation that non-sulfur-containing muscimol does not prevent inflammasome activation, suggested that the presence of the sulfur atom plays a key role in this activity. Due to this apparent importance of the sulfur group, here we examined other similar sulfur-containing molecules, taurine and isothiazole. We found that the presence of sulfur alone, including in the context of the core heterocyclic ring (isothiazole), is insufficient for preventing inflammasome activation, indicating that other molecular features also contribute.

Thiomuscimol is a potent inflammasome inhibitor, but thiomuscimol and muscimol both bind to the GABA-A receptor, producing additional effects that preclude them from being therapeutically applicable as inflammasome inhibitors in their current forms. A clinically relevant formulation could be developed by identifying the portions of the structure that specifically mediate inflammasome inhibition. To better understand the importance of the different structural elements in thiomuscimol that facilitate inflammasome inhibition, we analyzed sulfur-containing analogs of thiomuscimol containing changes in the primary amine substituent. We found that replacement of the primary amine with a carbonyl group (analog 1) abolished activity, but substitution with a secondary amine (analog 2) caused no appreciable change in inhibitory activity. Analog 2 inhibited inflammasome activation and subsequent pyroptotic pore formation and loss of cellular ATP, with similar potency to thiomuscimol. Replacing the secondary amine with an annulated piperidine ring (analog 3) reduced potency compared to thiomuscimol with an altered dose–response curve. Activity was sensitive to the position of the amine group within the piperidine ring structure, as analog 4 was inactive.

These insights into the structure–activity relationship will inform future experiments to better characterize the molecular mechanism that enables broad-spectrum inflammasome inhibition. Our present findings focus on NLRC4 and NLRP1b, but given thiomuscimol’s inhibition of other inflammasomes such as NLRP3 [15], future experiments could also examine the effect of analogs against other inflammasomes. Our previous finding that thiomuscimol activity is reversible [15] suggests that irreversible or covalent target binding or modification may not occur; however, further studies to identify the specific molecular mechanism of action are needed. In addition, structural modifications that increase potency are an area that warrants future study. The structural changes within the scope of our study were focused around the amine group, and an area of future direction would be extending the structural analyses of changes to the thiomuscimol molecule to characterize additional requirements. Such changes could include examining further permutations of the amine spacing within the ring structure, or adjusting the structure around the acidic hydroxyl group, acetylation of the nitrogen, or generation of tertiary amines. These experiments could provide further insight into whether protonation, hydrogen bond donation, or other physicochemical properties are critical for activity. Total replacement of the amine group, either within existing alterations such as the piperidine ring, or within the base thiomuscimol structure, would explore the impact of further altering the polarity and hydrogen bonding behavior on observed activity. This examination could provide additional insight into the exact requirements for inflammasome inhibition. The identification of a precise molecular target as a result of future analyses may clarify whether the biological response relies on hydrogen bond donating behavior, or specific interactions involving the sulfhydryl group of free cysteine residues that may have been altered or interrupted by some of the structural modifications present in our analogs.

## 4. Materials and Methods

### 4.1. Cell Culture

Bone marrow-derived macrophages (BMDMs) were cultured from femur exudates of both male and female mice from the mouse strains BALB/cJ (Strain #:000651), C57BL/6 J (Strain #:000664), and C57BL/6 J ASC–citrine (Strain #:030744), all purchased from Jackson Laboratories (Bar Harbor, ME, USA).

BMDMs were grown for 7 days at 37 °C with 5% CO_2_ in Dulbecco’s Minimal Essential Medium (DMEM) supplemented with 10% Serum Plus II (Sigma, St. Louis, MO, USA), 5 mM Hepes, 0.05 mM β-mercaptoethanol, 100 U/mL penicillin and streptomycin, and 30% conditioned medium from L929 cells (ATCC Cat. No CCL-1, RRID:CVCL_0462). Additional medium was provided on day 3 or 4. Cells were collected by washing with ice-cold PBS containing 1 mM EDTA, resuspended in 10 mL of supplemented medium without phenol red containing 5% Serum Plus II, and counted. For 96-well plates, cells were diluted to 4 × 10^5^ cells/mL and were seeded at 100 μL/well. For 384-well plates, cells were diluted to 8 × 10^5^ cells/mL, and were seeded at 50 μL/well. As primary cells were used immediately after differentiation and not maintained in sub-culture, cell line validation and mycoplasma testing were not performed.

### 4.2. Pyroptosis Inducers and Reagents

**Figure 5:**
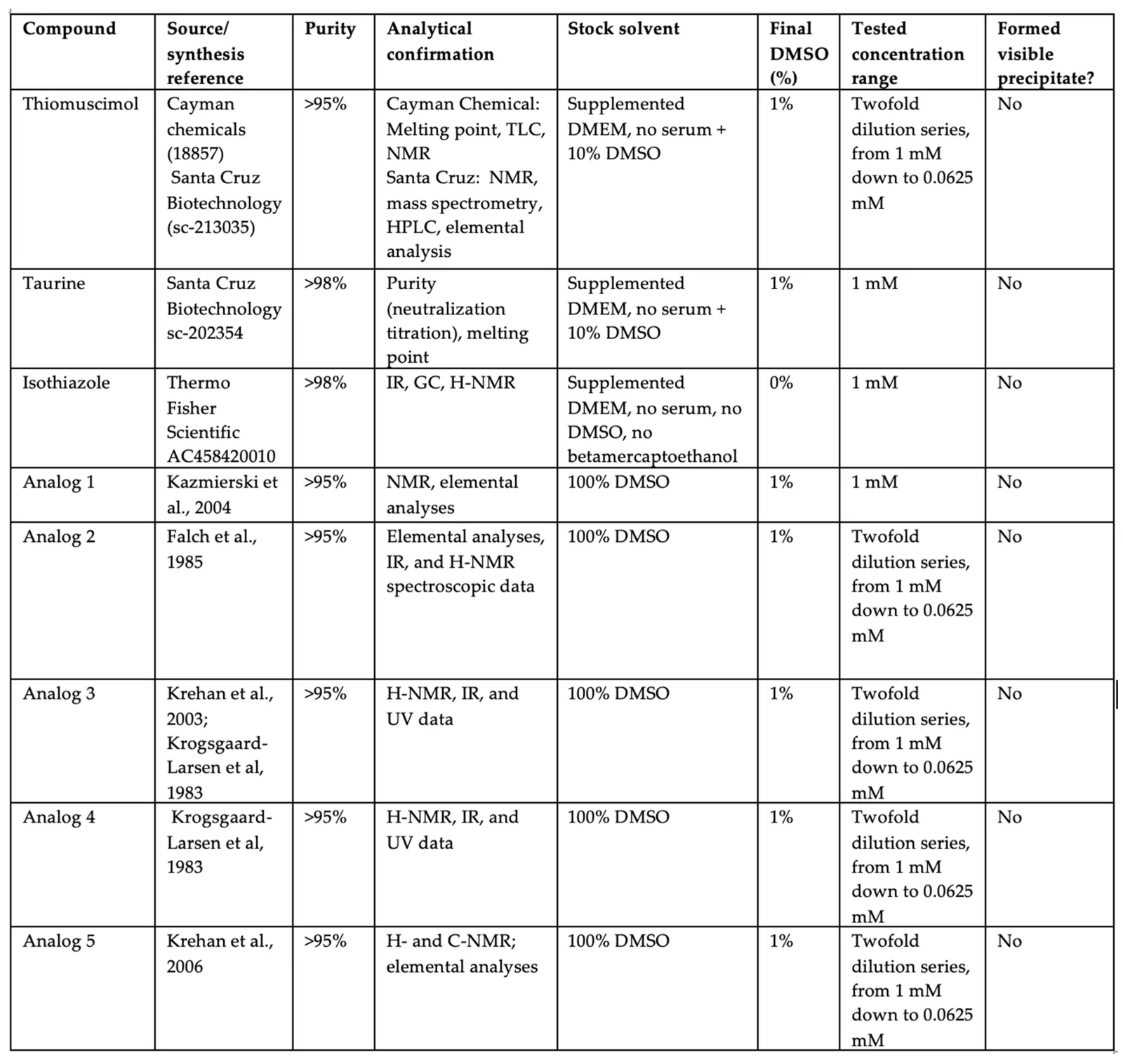
Summary of compounds used in this study. References used in this figure are: [23,24,25,26,27].

To activate the NLRC4 inflammasome, late-log cultures of *Salmonella enterica* serovar Typhimurium strain SL1344 were grown in Luria–Bertani broth containing 0.3 M NaCl with shaking at 37 °C. Bacteria were washed, resuspended in PBS, and added at a multiplicity of infection of 10 bacteria per macrophage for 2 h (unless otherwise indicated). To activate the NLRP1b inflammasome, BMDMs from BALB/c mice were exposed for 2 h to 1 μg/mL anthrax lethal toxin prepared as a 1:1 mix of protective antigen and lethal factor (List Biological (Campbell, CA, USA)). Taurine (Santa Cruz Biotechnology (Dallas, TX, USA)) and thiomuscimol (Santa Cruz Biotechnology and Cayman Chemicals (Ann Arbor, MI, USA)) were reconstituted in DMEM containing 10% DMSO. Isothiazole (Fisher Scientific (Waltham, MA, USA)) stock (and thiomuscimol stock for this experiment and replicates) was prepped in DMEM without β-mercaptoethanol. Thiomuscimol analogs were synthesized as previously described in the literature: analog 1 [23], analog 2 [24], analog 3 [25,26], analog 4 [26], and analog 5 [27]. Analogs were reconstituted in 100% DMSO. Thiomuscimol, taurine, and thiomuscimol analogs were diluted to their final concentrations, with all conditions for these experiments suspended in DMEM containing 1% DMSO. All compounds were added at specified concentrations at the same time as the pyroptosis inducer. Additional compound information is summarized in Figure 5.

### 4.3. Cellular ATP Measurement

Cellular ATP content was assessed using the luminescent Cell Titer Glo 2.0 Cell Viability Assay kit (Promega (Madison, WI, USA)), according to the manufacturer’s protocol.

### 4.4. Lactate Dehydrogenase (LDH) Release Assay

The cytotox 96 Non-Radioactive Cytotoxicity Assay kit (Promega) was used to measure plasma membrane rupture, according to the manufacturer’s protocol. Maximum LDH release was obtained by using the provided detergent-based lysis buffer. OD490 was measured using a Spectramax M3 plate reader (Molecular Devices, San Jose, CA, USA). LDH release was calculated as: 100 × (experimental LDH-spontaneous LDH)/(maximum LDH-spontaneous LDH).

### 4.5. IL-1β Enzyme-Linked Immunosorbent Assay (ELISA)

BMDMs were seeded in a 96 well plate, and then were primed with LPS (100 ng/mL, List Biological) for 3 h before removal of priming media and addition of media with and without compounds. Appropriate vehicle controls were used to match the media content of the different compounds (with and without 1% DMSO, or betamercaptoethanol). Released IL-1β levels were determined by ELISA, using the Quantikine ELISA kit (R&D Systems (Minneapolis, MN, USA) according to manufacturer’s protocol.

### 4.6. Live Cell Fluorescence Microscopy

BMDMs were seeded in optical 384-well plates and treated with pyroptosis inducers, with and without compounds. To assess ASC foci formation, cells expressing ASC-fusion protein were used. Syto13 dye (ThermoFisher (Waltham, MA, USA)) was used at 0.5 μM to count cells that do not express the ASC-fusion protein. Membrane impermeable nuclear dye To-PRO-3 (ThermoFisher) was used at 1 μM to assess gasdermin-D pore formation. ASC foci formation, Syto13 counts, and To-PRO-3 uptake were detected using appropriate channels (ASC foci = YFP, Syto13 = GFP, To-PRO-3 = Texas Red), and counts of each per time point were determined with signal detection for each color channel. Detection and quantification was done using a Cytation1 imaging system running Gen5 version 3.17 (Agilent BioTek (Santa Clara, CA, USA)). Percent To-PRO-3 uptake or percent foci formation was calculated by subtracting background counts, and then calculating the percentage of positive cells per well at each time point. Data presented are the percentage at the last time point for each experiment.

### 4.7. Statistics and Reproducibility

Appropriate statistical analysis was performed in Prism (Boston, MA, USA) (GraphPad software, v.10). The statistical tests used and the number of replicate wells are indicated in each figure legend, and data presented are representative of at least two separate experiments which were conducted with independent BMDM preparation. All data are presented as mean ± SD unless otherwise specified. All graphs were prepared in Prism (GraphPad software, v.10).

## Data Availability

The original contributions presented in this study are included in the article/Supplementary Material. Further inquiries can be directed to the corresponding author(s).

## Supplementary Materials

The following supporting information can be downloaded at: https://www.mdpi.com/article/10.3390/ijms27167235/s1.

## Abbreviations

The following abbreviations are used in this manuscript:

BMDMs	bone marrow-derived macrophages
CARD	caspase recruitment domain
IL-1β	interleukin-1β
PYD	pyrin domain
