# Supplementary material for: Structure–Activity Relationship for Inflammasome Inhibition by Thiomuscimol
Source: Int J Mol Sci. 2026 Aug 13;27(16):7235. doi: 10.3390/ijms27167235 (PMC13513477; doi:10.3390/ijms27167235)
Supplement: Supplementary file 1 [file ijms-27-07235-s001.zip › ijms-4385404-supplementary.pdf]

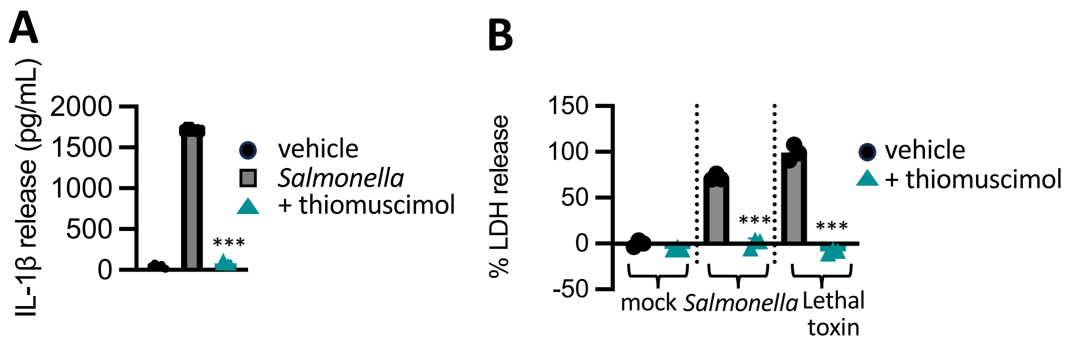

Supplemental Figure S1. Thiomuscimol inhibits IL-1β release and LDH release during pyroptosis.
BMDM were infected with *Salmonella* at an MOI of 10 (A-B) or treated with lethal toxin (B) to induce pyroptosis in the presence of the indicated compounds. Supernatant was collected, and IL-1β release was analyzed (A), or LDH release was analyzed (B). Data are means +/- SD, n=5 replicate wells (A), n=3 replicate wells (B) and are representative of two independent experiments. Stars (*) indicate statistical significance against *Salmonella* or lethal toxin. *** P<0.001(One way ANOVA, with Tukey's multiple comparisons test).

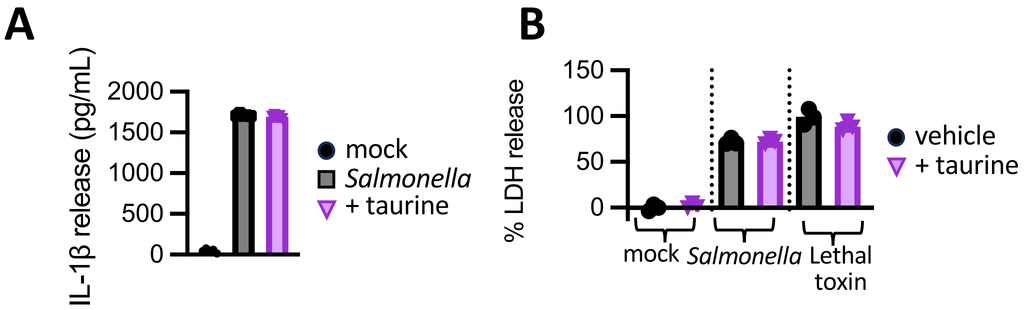

Supplemental Figure S2. Taurine does not inhibit IL-1β release or LDH release during pyroptosis.
BMDM were infected with *Salmonella* at an MOI of 10 (A-B) or treated with lethal toxin (B) to induce pyroptosis in the presence of taurine as indicated. Supernatant was collected, and IL-1β release was analyzed (A), or LDH release was analyzed (B). Data are means +/- SD, n=5 replicate wells (A), n=3 replicate wells (B) and are representative of two independent experiments.

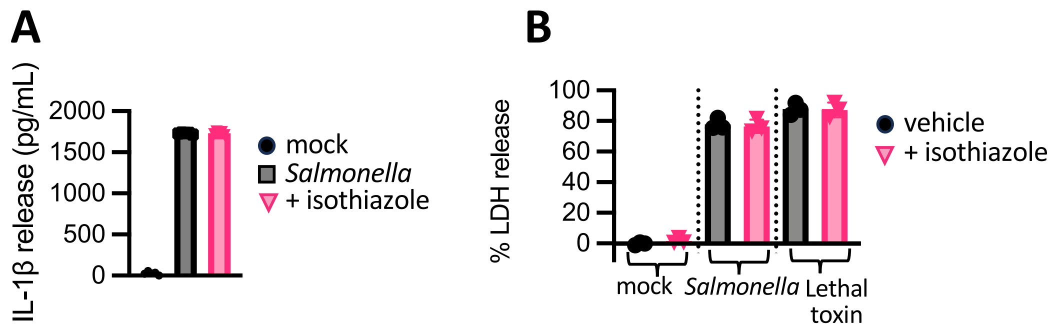

Supplemental Figure S3. Isothiazole does not inhibit IL-1β release or LDH release during pyroptosis.
BMDM were infected with *Salmonella* at an MOI of 10 (A-B) or treated with lethal toxin (B) to induce pyroptosis in the presence of isothiazole as indicated. Supernatant was collected, and IL-1β release was analyzed (A), or LDH release was analyzed (B). Data are means +/- SD, n=5 replicate wells (A), n=3 replicate wells (B) and are representative of two independent experiments.

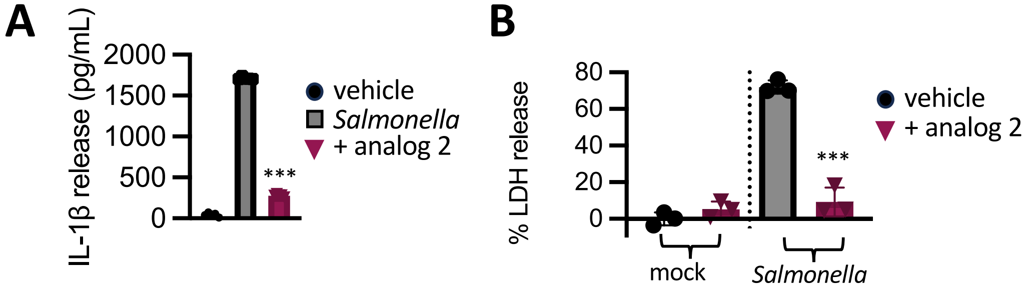

Supplemental Figure S4. Analog 2 inhibits pyroptosis-induced IL-1β release and LDH release.
BMDM were infected with *Salmonella* at an MOI of 10 to induce pyroptosis in the presence of the indicated compound. Supernatant was collected, and IL-1β release was analyzed (A), or LDH release was analyzed (B). Data are means +/- SD, n=5 replicate wells (A), n=3 replicate wells (B) and are representative of two independent experiments. Stars (*) indicate statistical significance against *Salmonella*. *** P<0.001(One way ANOVA, with Tukey's multiple comparisons test).

A

analog 5

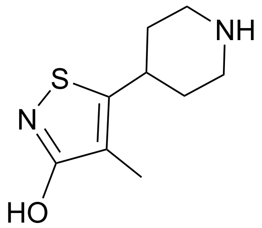

B

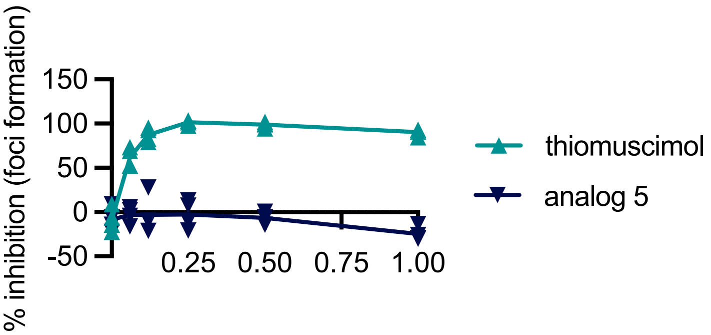

Supplemental Figure S5. Non-annulated piperidine ring eliminates inhibitive activity. Structure of analog 5 (A). BMDM were infected with *Salmonella* at an MOI of 10 to induce pyroptosis in the presence of the indicated compounds. ASC foci formation was assessed and inhibition at the indicated concentration was calculated relative to vehicle control (B). Data are means +/- SD, n=3 or 4 replicate wells, and are representative of at least two independent ex-periments.
